# Evaluation of psychosocial aspects in participants of cancer genetic counseling

**DOI:** 10.1186/s13053-017-0073-x

**Published:** 2017-09-20

**Authors:** Leivy Patricia González-Ramírez, Reyna Martínez-Arriaga, Erendira Camacho-Cárdenas, Azucena Del Toro-Valero, Antonio Oceguera-Villanueva, Livia Zagamé, Aída Araceli Silva-García, Adrián Daneri-Navarro

**Affiliations:** 1 Departamento de Ciencias de la Salud, Centro Universitario de Tonala, Universidad de Guadalajara, 555 Nuevo Periférico Av. Ejido San Jose Tatepozco, 45425 Guadalajara, Jalisco Mexico; 2 Programa de Asesoramiento Genético Oncológico, Centro Universitario de Ciencias de la Salud, Universidad de Guadalajara, 950 Sierra Mojada St., Independencia, 44340 Guadalajara, Jalisco Mexico; 3 Instituto Jalisciense de Cancerología, Secretaría de Salud Jalisco, 715 Coronel Calderón St., El Retiro, 44280 Guadalajara, Jalisco Mexico; 4 O.P.D. Hospital Civil Nuevo de Guadalajara “Dr. Juan I. Menchaca”, 876 Salvador Quevedo y Zubieta St., Independencia Oriente, 44340 Guadalajara, Jalisco Mexico; 5 Departamento de Fisiología, Centro Universitario de Ciencias de la Salud, Universidad de Guadalajara, 950 Sierra Mojada St., Independencia, 44340 Guadalajara, Jalisco Mexico

**Keywords:** Cancer genetic counseling, Breast cancer, Anxiety, Depression scale, Psychological Health Interview

## Abstract

**Background:**

The instrument called “Hospital Anxiety and Depression Scale” (HADS) is frequently used to evaluate anxious and depressive symptomatology in patients who receive Cancer Genetic Counseling (CGC). However, this instrument cannot identify all of the psychosocial factors, such as the antecedents of the patients’ emotional states or their concerns. The objective of the present research was to compare cases detected with psychosocial alterations by means of HADS and a Psychological Health Interview (PHI).

**Methods:**

A transversal analytical design was used. One hundred ten participants were included (97.3% females and 2.7% males). The average age was 45 years ±10 years.

**Results:**

The PHI identified twice the amount of participants with psychosocial alterations than did HADS, which only detected 43% of these participants.

**Conclusions:**

The results of our study suggest that the PHI should be applied in addition to HADS to identify participants who would require psychological support due to recurrent concerns.

## Background

There are gene mutations that predispose cancer development, such as the BRCA 1 and BRCA 2 genes, which are associated with malignant tumors of breast and ovary [1]. Recently it has been described that mutations in these genes explain only 25% of cases of Hereditary Breast and Ovarian Cancer (HBOC). At least another 25 genetic factors in addition to BRCA 1/BRCA 2 predispose to HBOC. These factors are represented by genes that participate in DNA repair pathways, replication stability and checkpoints of genetic damage [2]. However, the presence of mutations in BRCA 1/BRCA 2 genes does lead to a high risk to develop cancer [3]. BRCA 1 mutation carriers have up to 40% of risk for ovarian cancer. Moreover, in BRCA 2 carriers, the risk increases up to 20% [4]. The study of these mutations in populations at risk should be accompanied by Cancer Genetic Counseling (CGC), which includes an evaluation process directed by experts. The purpose of the evaluation is to identify and advice CGC participants regarding the risk for cancer, including preventive and prophylactic measures. Discussions are conducted on motivations, expectations and the understanding of the genetic test results in order to support subsequent decision-making [5].

Anxiety and depression are two of the most studied psychological factors in participants of CGC [6, 7]. Since approximately 25% of participants who attend CGC experience clinically significant levels of anxiety, with recurrent concerns as one of the most characteristic symptoms of this illness [8]. It has been reported that low social support and personal/familial history of cancer are risk factors for anxiety [9]. Anxiety diminishes over time without disappearing [10] and persists in those with positive genetic results [11]. Other factors that influence the presence of anxiety are ambiguous communication with health professionals [12] and the informative method used [13]. Anxiety levels have been associated with decision-making, compliance to screening methods and to risk-reduction measures [14]. However, we did not find similar studies in Mexican population.

Assessments of psychosocial aspects during CGC are usually carried out by genetic counselors, nurses or social workers, using tests such as the Hospital Anxiety and Depression Scale (HADS) [15, 16]. However, this scale is not sufficient for detecting additional psychosocial implications in CGC, such as worry about cancer and inheriting cancer, fears, self-esteem, familial problems and social problems [17, 18]. Besides, Eijzenga et al. [18], note that HADS is too general to evaluate the psychosocial implications presented in the Genetic Counseling Services. These authors created the Psychosocial Aspects of Hereditary Cancer Questionnaire (PAHC) in Netherlands, which addresses themes that were absent in the instruments employed in genetic counseling services. We created the Psychological Health Interview (PHI) due to lack of this kind of instruments validated in our Mexican population. The PHI was constructed according the needs of our population as well as the Mexican Health System. The PHI allows identifying the previously mentioned implications, especially when the interviews are applied by psychologist. PHI is based on the premise that disease is influenced by psychosocial and behavioral variables such as emotional states, lifestyles, habits, culture, myths, familial variables and others [19]. The purpose of the present research was to compare HADS versus PHI in Mexican population in order to demonstrate the advantage of PHI in combination with HADS.

## Methods

### Participants

From July 2013 through January 2016, 180 participants referred by the Instituto Jalisciense de Cancerologia, Hospital Civil Nuevo de Guadalajara “Dr. Juan I. Menchaca” and some private hospitals, were initially evaluated by a geneticist at the Cancer Genetic Counseling Program of the Universidad de Guadalajara. The geneticist confirmed that the participants complied with HBOC inclusion criteria. A blood sample was drawn from each participant to detect possible mutations in BRCA 1, BRCA 2, as well in other high-risk genes for HBOC. Later, these participants were referred to the psychology service for a complete evaluation. Of the total of patients seen in the program, only one hundred ten patients (61%) kept their appointments with the psychologist. The principal reasons referred by patients for not keeping their appointments, were lack of time or lack of interest in a psychological follow-up. In addition, some patients deceased or were unable to be contacted to set up their appointment.

### Procedure and instruments

The instruments utilized by the psychology service were applied personally during the first appointment by a psychologist trained in hereditary cancer variables. The time required to complete the evaluation ranged from 40 min to 1 h. The evaluation included a Psychological Health Interview (PHI), which explores the psychosocial aspects that influence the patients’ health. This is a semi-structured interview, created through an extensive review of the literature and with the consensus of three experts in the area of the health psychology, psychooncology and genetic counseling who are part of the cancer genetic counseling program team. The interview consisted of 40 questions and contained the following areas: familial, social, health, psychological, perception of and information on cancer, lifestyle and cancer genetic counseling. Some examples of the questions in our PHI are presented in Table 1. The questions considered in the present publication were extracted from the psychological area and were as follows: 1) Have you experienced some loss or grief during the last 5 years (death, divorce, loss of employment, etc.)? In the affirmative case, please specify; 2) is there something that worries you in a recurrent manner at present? In the affirmative case, specify; 3) has this worry caused you sleep problems?

**Table 1:** Examples of questions in the psychological health interview

Issue	Question
Familial	How often do you talk with your family about your worries about cancer? (Choose number 1 to 10, where 1 is “Not at all” and 10 is “very often”)
Social	Do you have friendships that are important for you?
Health	Have you been diagnosed with cancer? How long ago? Which treatments have you received?
Psychological	Have you ever consulted a psychologist or a psychiatrist?
Perception of and information on cancer	What comes to mind when you hear the word cancer?
Lifestyles	Do you smoke or have you ever smoked?
Cancer Genetic Counseling	What advantages and disadvantages do you find in Cancer Genetic Counseling?

Also the Hospital Anxiety and Depression Scale (HADS) was applied. This is an instrument that comprises 14 items: seven to measure anxiety and seven to measure depression experienced in the previous week. Response options were scored from 0 to 3. In each sub-scale, scores of >10 indicate morbidity, scores between 8 and 10 are interpreted as borderline and scores of <8 normal. The HADS was validated in Mexican population with a reliability of 0.81 for anxiety and 0.82 for depression [20]. Anxiety items evaluate whether the person has been worried or has experienced sensations of fear, while depression items evaluate anhedonia or pessimistic thoughts [8].

### Ethical considerations

The evaluations and procedures performed within the Genetic Counseling Program of the Universidad de Guadalajara form part of a research project entitled “Molecular Genetic Studies for Patients with Cancer and Their Relatives” which is conducted in collaboration with the previously mentioned hospitals and City of Hope National Medical Center in the U.S. The project has the approval of the Committees of Investigation, Ethics, and Biosafety of all participating institutions: Instituto Jalisciense de Cancerología (004/12), O.P.D. Hospital Civil Nuevo de Guadalajara “Dr. Juan I. Menchaca” (1289/13) and the Universidad de Guadalajara (C.I. 035–2011). Informed consent was obtained from all of the participants included in the study. This consent mentioned the performance of the genetic test and the clinical and psychological evaluations. The participants were also informed of the research goals.

### Statistical analysis

For statistical analysis, we utilized the Statistical Package for Social Sciences (SPSS) v. 20.0 software program. We obtained frequencies, percentages, mean and Standard Deviation (SD) to describe the sociodemographic and clinical characteristics.

A dichotomous variable was generated for the HADS anxiety and depression sub-scale, grouping as “With anxiety” or “With depression”, including “borderline” and “comorbidity” categories.

Similarly, we included a dichotomous variable to register responses 1, 2 and 3, previously described in the psychological area of our PHI, to determine whether there existed or not recent grief, recurrent worries, or sleep problems. Worry types were categorized as follows: 1) health or genetic result related; 2) non health or non genetic result related, such as familial, economic and employment problems; and 3) multiple worries encompassing both categories.

We employed the Spearman correlation to determine a relation between the variables of the two instruments. Finally, we carried out a Chi-squared test and a Fisher Exact test to compare the cases detected in each of the HADS sub-scales vs. cases with psychosocial implications identified by PHI.

## Results

Regarding the sociodemographic characteristics, 107 of the total participants were women (97.3%). Average age was 45 years ± a SD of 10 years, 77 were married (60.9%) and 80 resided in the Metropolitan Zone of Guadalajara City (72.8%). With respect to scholarship, 21 patients had 6 years or less of study (19.1%), 55 had 7–12 years (50%), and 32 patients more than12 years (29.1%). Only 43 patients had employment (39.1%), while the remainders were unemployed, homemakers, retired persons or students (60.9%). Patients with personal history of cancer were 89 (80.9%), mainly diagnosed with breast cancer (91%). Average age at the time of diagnosis was 39 years (± an SD of 8.5 years).

HADS detected only 31 patients with anxious symptomatology (29.1%), while 17 manifested depressive symptoms (15.5%). In contrast, our interview detected 72 patients with recurrent worries (65.5%), with non health-related worries (*n* = 30; 41.7%) and health-related worries (*n* = 26; 36.1%). One third of these patients experienced sleep problems. Of this last group, 5 participants (20.8%) reported health-related worries; 10 participants (41.67%) had non-health-related worries and 6 (25%) with both categories. Based on our PHI, we found that 77 (60.9%) patients had experienced a grief during the last 5 years.

**Fig. 1:**
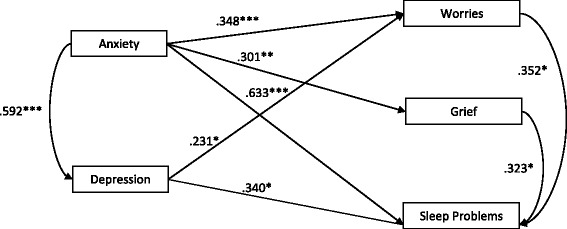
Correlations between anxiety and depression obtained by HADS with respect to worries, grief, and sleep problems obtained with the PHI. Note: Statistical significance = *p < 0.05, **p < 0.01, and ***p < 0.001

The results demonstrated that there was a correlation between the variables detected by HADS and those identified by our PHI (Fig. 1). Anxiety had a significant correlation with worries (*p* = 0.001; *r* = 0.348), sleep problems (*p* < 0.001; *r* = 0.633), and grief (*p* = 0.004; *r* = 0.301). Meanwhile, depression correlated with worries (*p* = 0.031; *r* = 0.231) and sleep problems (*p* = 0.019; *r* = 0.340).

We observed that PHI identified more psychological implications than HADS (Tables 2 and 3). We found a significant difference in anxiety evaluated by HADS versus recurrent worries (*p* = 0.001), type of worry (*p* = 0.042), grief (*p* = 0.004) and sleep problems (*p* < 0.01) evaluated by PHI. Also, in depression evaluated by HADS, we appreciated a significant difference with recurrent worries (*p* = 0.034).

**Table 2:** Comparison of anxiety and depression sub-scales (HADS) versus recurrent worries and grief referred in the psychological health interview (PHI)

	Recurrent worries			Grief in the past 5 years		
	Yes	No	*P*	Yes	No	*P*
Anxiety						
No	41 (57%)	16 (100%)	0.001*	38 (57%)	19 (90%)	0.004*
Yes	31 (43%)	0 (0)		29 (43%)	2 (10%)	
Depression						
No	55 (76%)	16 (100)	0.034*			
Yes	17 (24%)	0 (0)				

Note: Table shows frequencies and percentages. Anxiety and depression sub-scales obtained from the HADS; Recurrent worries and grief in the past from PHI

*P value obtained with the Fisher Exact statistical test

**Table 3:** Comparison of the anxiety sub-scale obtained of HADS versus type of worry and sleep problems of PHI in participants in cancer genetic counseling

	Anxiety		*P*
	No	Yes	
Type of worry			
Health-related	19 (46%)	7 (25%)	0.042*
Non-health-related	18 (44%)	12 (43%)	
Both	4 (10%)	9 (32%)	
Sleep problems			
Yes	7 (25%)	17 (89%)	<0.001*
No	21 (75%)	2 (11%)	

*P value obtained with the Chi-squared statistic

## Discussion

It has been demonstrated that the application of HADS in combination with questionnaires or interviews, provides greater understanding and sensitivity to identify psychosocial alterations in CGC participants [18]. Our results showed that the combined use of PHI with HADS allowed us to identify a greater number of psychosocial alterations in CGC participants, without losing the benefits of the standardized measurement of anxiety and depression provided by HADS. We found a correlation between the variables evaluated by HADS and PHI. Our results suggest that anxious and depressive symptomatology, worries, grief, and sleep problems affect the well-being of the CGC participants. In addition, according to Nogueda Orozco and collaborators [8], recurrent worries are one of the most common symptoms in persons who experience anxiety.

Despite the correlation between the variables evaluated by HADS and PHI, we observed differences in the number of participants detected with psychosocial implications. HADS detected 31 patients with anxiety, while PHI identified 72 patients with recurrent worries. HADS retains its use as a screening instrument for detection of anxious and depressive symptomatology and is one of the most used in this population [18]. In spite of the importance of this scale, our results and those of Eijzenga et al. [18], suggest that HADS is too general for encompassing the broad spectrum of relevant psychosocial variables presented in the Genetic Counseling Services.

Our results highlight the relevance of combined use of PHI and HADS to identify psychosocial needs on CGC participants.

It is well known that anxiety varies depending on the sociocultural aspects of the population, ranging from 16% to 66% [21]. Our results suggest that the number of cases with anxiety may be underestimated with the single use of HADS. Moreover, it has been reported that anxiety is present before and after the genetic study [22, 23]. Thus, it is indispensable to evaluate systematically and in detail the psychosocial variables during the CGC process. Hirschberg and collaborators [22], suggest the evaluation of psychosocial variables during the genetic counseling process in order to refer patients for psychosocial interventions.

According to our results 38% of recurrent worries were closely linked with self and family health, such as worries about cancer recurrence or the finding of genetic mutation. In addition, 43% of the patients reported non health-related worries, such as personal, employment, or familial issues. Nearly 19% of the participants referred both health and non-health worries. It has been reported that non-health worries are not evaluated in CGC, due to the screening of emotional implications performed by physicians, nurses or geneticists [15, 24, 25].

On the other hand, our study revealed that one third of participants who expressed worries through the PHI had also sleep problems. This problem has been reported in oncological patients [26, 27], as well as in BRCA carriers after prophylactic salpingo-oophorectomy [28]. However, there are not reports regarding the prevalence of sleep problems during the CGC. It is relevant to study this aspect, since it has been reported that it is associated with anxiety, depression [29] as well as poor perception of health and a low quality of life [30].

In this study, we found a higher frequency of depressive symptomatology in our studied population (15.5%) in comparison with other studies of CGC participants (4–5%) [31]. This could be due to sociocultural differences, but this hypotheses should be confirmed in future studies. Given that HADS only evaluates depressive symptomatology, it does not sustain a formal diagnosis of depression. Therefore, we suggest a diagnostic evaluation based on criteria of the Diagnostic and Statistical Manual of Mental Disorders V and consequently appropriate management [32]. Our PHI, also evaluates psychopathological antecedents and the use of drugs. Other studies suggest the evaluation of psychosocial aspects before genetic counseling to decide the best time to receive the CGC. It is well known that anxiety diminished after CGC, but this did not occur with depression [14]. In addition, an opportune psychological intervention benefits patients with depression or anxiety prior to the genetic study [14].

In our experience, patients who require psychological follow-up, due to non health-related worries or genetic counseling, are referred to the Psycho-Oncology service or Patient Navigation Program for Cancer Patients and their Relatives, to maximize the available institutional resources and alliances.

### Study limitations

Our study is ongoing with more than 100 patients, but is under study other 200 participants and the corresponding follow-up.

The study design is cross-sectional and lacks the possible differences over time.

### Recommendations and next steps

We recommend the application of HADS and PHI to identify psychosocial needs in participants of the Cancer Genetic Counseling Program. Genetic Counselors, physicians, nurses or other professionals with training in CGC and psychosocial issues could use the PHI. According with our experience, PHI is applied by psychologists, who offer not only the evaluation of psychosocial implications, but also support adjusted to each participant.

It is important to compare the PHI with other instruments such as the Impact of Events Scale, Perceived Stress Scale and the Center for Epidemiologic Studies Depression Scale in order to have objective information about the utility and pertinence in CGC programs.

It is important to follow-up psychological aspects in both mutation carriers and non-carriers.

We need to know whether family deaths are due to other causes and their consequent influence on the psychological well-being and decision making of patients.

## Conclusions

The PHI allows identify important psychosocial aspects into CGC participants. The combined use of HADS and PHI offer more advantages. The PHI could be used by any health professional into CGC, especially by a psychologist with training in CGC. The accurate evaluation and management of the emotional aspects will contribute to achieve the objectives of the CGC services. Informed decision-making, better communication and genetic counseling for family members with high risks for cancer would best conform to medical recommendations.
